# A Practical Point in the Use of Disinfectants and Antiseptics

**Published:** 1895-10

**Authors:** A. G. Young

**Affiliations:** Augusta, Maine.


ARTICLE V.

### A PRACTICAL POINT IN THE USE OF DISINFECTANTS AND ANTISEPTICS.

BY A. G. YOUNG, M. D., AUGUSTA, MAINE.

In the large mass of literature which has appeared on the same subject since Koch's paper, "On Disinfection," it is rather surprising that the practical value of one of the suggestions in that paper was not more promptly apprehended and worked out. It was contained in this paragraph:

"Yet it is probable that many disinfecting agents which, under ordinary circumstances are inefficient, may become sufficiently active when combined with moderately increased temperatures; possibly also some such substances which have no disinfecting action at all at temperatures in the neighborhood of 20° C. (68° F.), as the example of carbon bisulphide teaches, may be used with excellent results at somewhat higher temperatures. In this direction, then, is a field open which will reward experimental activity, and which all the more deserves attention because exact experiments are wanting with all but a very few af the greater number of disinfectants and these have been shown to be practically useful only under certain conditions."*

In 1889, Professor Scalji,† of Rome, mentioned the following Significant facts:

Normal urine, when maintained at a temperature of 45° C. (113° F). undergoes fermentation as readily as when left at a temperature of from 15° to 25° (59° to 77°F.). If

*Mittheilungen aus dem Kais. Gesundheitsamte, 1, 249, 1881.

†Bulletin Medical—Revue D Hygiene, XII, 82. 1880.

5 centigrams of corrosive sublimate are added to one litre of urine, making a solution of 1: 20,000, the urine putrefies at the end of several days if left at ordinary temperatures; but it is preserved a month or more without a trace of fermentation if its temperature is maintained at 40° (104° F.). At this temperature of 40° the antiseptic action is assured with a dose of only 1 : 100,000 of the sublimate.

Furthermore, Professor Scalji states, that when urine which contains 1:100,000 of sublimate is kept at the temperature of 40° several days, and then allowed to remain at ordinary temperatures (15° to 20° C.), no trace of fermentation appears.

Behring* refers to the fact, which has been confirmed by various observers, that a 5 per cent. solution of carbolic acid at ordinary room temperature will not, even after many days, destroy anthrax spores with certainty, but he shows that, when the temperature of the solution is raised to 37.5° C. (99.5° F;), their destruction is complete in three hours.

Caustic solutions of the alkalies are more or less rapidly germicidal according to their strength, but Behring learned that the alkaline carbonates may become very energetic disinfectants when used at higher than ordinary temperatures. After working with strong solutions of carbonate of soda and of alkaline soaps, and finding that they, at degrees of temperature above 70° and 80° (158° and 176° F.), were rapidly effective, made a solution of washing soda about as it is used in laundries, and containing about 1.4 per cent. of soda. This solution at 80°—83° C. (176° — 181.4° F.) destroyed anthrax spores in ten minutes, and at 75° C. (167° F.), in twenty minutes.

He confesses that these results with warm and hot solutions of washing soda surprised him, particularly as he had, through special control experiments, assured himself of the high powers of resistance which his anthrax spores possessed.

*Zeitschrift fur Hygiene, IX, 395. 1890.

But, so far as I know, Heider* has marked out more fully than any one else the influence which moderately increased temperature has upon the action of disinfecting solutions. Among the results of his investigations we may note the following, in which anthrax spores served as a test and the number of minutes, hours, or days given, being the time required to sterilize them.

Carbolic acid, 5 per cent. solution, at ordinary room temperature, not destroyed from thirty to forty days, at 40° C. (104° F.), four hours; at 55° C. (131° F.) from three-fourths to two hours; at 75° C. (167° F.), from three to fifteen minutes.

Pure carbolic acid and sulphuric acid, equal parts of each by weight, 5 per cent. solution, at 40°, in two hours; at 55°, in thirty minutes, at 75°, in one minute.

Pure cresol and sulphuric acid, equal parts of each, 5 per cent. solution, at 40°, in one hour; at 55°, in five minutes.

Lysol, 5 per cent. at 60° C. (140° F.), sterilization not effected in two hours; at 80° (176° F.), sterilization complete in five minutes.

Sulphuric acid, 1 per cent., at ordinary temperatures, sterilization not effected in seven hours; at 75° C., sterilization in seventy minutes.

Caustic potash. 5 per cent. solution, at temperature of room, failed to sterilize in eight to ten hours; at 55°, spores destroyed in three-fourths to two hours; at 75°, in two to ten minutes.

Hot water, at 70° (158° F.), in eight to nine hours; at 85° (185° F.), in forty to forty-five minutes; at 95° (203° F.), in fifteen minutes.

Sporeless bacteria, of course, succumb much more readily to the germicidal powers of disinfectants. With stophyloccus pyogenes aureus as a test, sterilization was complete at the temperature of 60° (140° F.), with carbolic acid, 1 per cent., in five minutes; with carbolic acid and

---

*Arcoiv fur Hygiene, XV, 341. 1892.

sulphuric acid, 1 per cent., in one minute; with caustic potash, 1 per cent., three minutes; with lysol, one-half per cent., three minutes.

Several explanations of the reason why warm solutions of disinfectants show a more energetic germicidal action than cold solutions, suggest themselves. One is the well-known fact that the intensity of chemical action increases with increasing temperature; another is that moderately elevated temperatures favor the functional activity of bacterial life and therefore the rapidity with which poisons are absorbed. But when we have to do with spore-less bacteria, and that is the case in nearly all of the real work of disinfection, we have the direct co operation of moist heat in destroying its vitality, even when the increase in temperature is hardly more than moderate.

Practical applications of the results of these investigations readily occur. In the first place, they suggest a grave doubt as to the efficacy of some processes of disin fection and antisepsis as they may be carried out during the cold season. Next, they teach the great advantage of using antiseptic and disinfecting solutions warm or even hot.

In the disinfection of material containing the bacillus of tuberculosis, we have to do with an infection hard to destroy, but about whose powers of resistance, whether against chemical disinfectants or heat, investigators differ. Schill and Fischer* state that twenty four hours are required for a 5 per cent. solution of carbolic acid to disinfect tuberculous sputum. But the disinfection may be more rapidly and certainly accomplished if the carbolic solution for the disinfection of clothing be heated, or if the spittoon containing sputum and disinfecting solution be filled with hot water and set aside to cool before it is emptied. Warm or hot solutions may therefore be used for the purposes of increasing the intensity of action of some of the disinfectants, thus extending the range of their applicability.

---

*Mitteilungen aus dem Kais. Gesundheitsamte II, 142. 1884.

When the articles to be disinfected can be subjected to the action of the solution for only a short time, as in washing floors or other woodwork, wiping down walls, or rubbing articles of leather or upholstered furniture which cannot be disinfected otherwise, rapidity and certainty of action should be increased by increasing the temperature of the disinfecting solution.

In clinical disinfection and antisepsis, it is an advantage to use the solutions as warm as practicable. It will sometimes enable us to diminish the strength of our solutions and thus minimize the danger of toxicity, without loss of disinfecting effect. In the dressing of wounds, and as intra-pleural and intra uterine injections Professor Scalji says that he has used with excellent results very dilute but very warm, bichloride solutions (40°—45° C.= 104°—113° F.).

Another point of advantage in the use of warm disinfecting solutions is made by Nocht.* In the use of the so-called 100 per cent. carbolic acid, its slight solubility in water is a hindrance. Mixed with water in the proportion of 5 : 100 a large portion of it remains undissolved. Clothing immersed in the mixture is permanently spotted and injured, for the reason that the fabric readily absorbs the insoluble part as well as the watery solution, and so is in places, subjected to the action of the undiluted agent. If, however, to a hot solution of soap and water, the carbolic acid is added, and the mixture stirred, a clear solution is formed. Three per cent. of soap and water at 60° (140° F.) will render 6 per cent of carbolic acid soluble. The appearance of fabrics soaked in this solution is much better than if subjected to the simple watery solution. The germicidal action of the solution is not increased by the presence of the soap, but heightened temperature makes it distinctly more effective. At 50° (122° F.) a 5 per cent. solution with soap destroyed anthrax spores in six hours.—*Journal of Medicine and Science.*

---

*Zeitschrift fur Hygiene VII, 521. 1889.

